# Using text mining techniques to extract prostate cancer predictive information (Gleason score) from semi-structured narrative laboratory reports in the Gauteng province, South Africa

**DOI:** 10.1186/s12911-021-01697-2

**Published:** 2021-11-25

**Authors:** Naseem Cassim, Michael Mapundu, Victor Olago, Turgay Celik, Jaya Anna George, Deborah Kim Glencross

**Affiliations:** 1 Department of Molecular Medicine and Haematology, Faculty of Health Sciences, University of Witwatersrand and National Health Laboratory Service (NHLS), 7 York Road, Parktown, Johannesburg, South Africa; 2 School of Public Health, Faculty of Health Sciences, University of Witwatersrand, 7 York Road, Parktown, Johannesburg, South Africa; 3 National Health Laboratory Service (NHLS), National Cancer Registry (NCR), 1 Modderfontein Road, Sandringham, Johannesburg, South Africa; 4 School of Electrical & Information Engineering and Wits Institute of Data Science, University of Witwatersrand, 1 Jan Smuts Avenue, Braamfontein, Johannesburg, South Africa; 5 Department of Chemical Pathology, Faculty of Health Sciences, University of Witwatersrand and National Health Laboratory Service (NHLS), 7 York Road, Parktown, Johannesburg, South Africa

**Keywords:** Prostate cancer, Gleason score, Late presentation, Text mining, Algorithm, Public health

## Abstract

**Background:**

Prostate cancer (PCa) is the leading male neoplasm in South Africa with an age-standardised incidence rate of 68.0 per 100,000 population in 2018. The Gleason score (GS) is the strongest predictive factor for PCa treatment and is embedded within semi-structured prostate biopsy narrative reports. The manual extraction of the GS is labour-intensive. The objective of our study was to explore the use of text mining techniques to automate the extraction of the GS from irregularly reported text-intensive patient reports.

**Methods:**

We used the associated Systematized Nomenclature of Medicine clinical terms morphology and topography codes to identify prostate biopsies with a PCa diagnosis for men aged > 30 years between 2006 and 2016 in the Gauteng Province, South Africa. We developed a text mining algorithm to extract the GS from 1000 biopsy reports with a PCa diagnosis from the National Health Laboratory Service database and validated the algorithm using 1000 biopsies from the private sector. The logical steps for the algorithm were data acquisition, pre-processing, feature extraction, feature value representation, feature selection, information extraction, classification, and discovered knowledge. We evaluated the algorithm using precision, recall and F-score. The GS was manually coded by two experts for both datasets. The top five GS were reported, with the remaining scores categorised as “Other” for both datasets. The percentage of biopsies with a high-risk GS (≥ 8) was also reported.

**Results:**

The first output reported an F-score of 0.99 that improved to 1.00 after the algorithm was amended (the GS reported in clinical history was ignored). For the validation dataset, an F-score of 0.99 was reported. The most commonly reported GS were 5 + 4 = 9 (17.6%), 3 + 3 = 6 (17.5%), 4 + 3 = 7 (16.4%), 3 + 4 = 7 (14.7%) and 4 + 4 = 8 (14.2%). For the validation dataset, the most commonly reported GS were: (i) 3 + 3 = 6 (37.7%), (ii) 3 + 4 = 7 (19.4%), (iii) 4 + 3 = 7 (14.9%), (iv) 4 + 4 = 8 (10.0%) and (v) 4 + 5 = 9 (7.4%). A high-risk GS was reported for 31.8% compared to 17.4% for the validation dataset.

**Conclusions:**

We demonstrated reliable extraction of information about GS from narrative text-based patient reports using an in-house developed text mining algorithm. A secondary outcome was that late presentation could be assessed.

## Background

Globally, prostate cancer (PCa) is an important non-communicable disease (NCD) due to both population growth and a concomitant increase in life expectancy [1, 2]. It is the leading male neoplasm in South Africa with an age-standardised incidence rate (ASIR) of 68.0 per 100,000 population in 2018 [3].

Local treatment guidelines indicate that men with PCa are assigned to risk categories using the prostate specific antigen (PSA) result, Gleason score (GS) and clinical stage [4, 5]. The GS is based on the predominant histological pattern noted across all prostate biopsy samples submitted for anatomical pathology (AP) review, with a score of 1 reflecting the presence of normal cells and incremental mutational (grade) malignant change reflected in a score of 2 to 5. Within the scoring system, the first GS reflects the predominant cell pattern whereas the second Gleason grading is determined by the second most predominant pattern. For example, a GS of 3/5 (primary or major) and 4/5 (secondary or minor) equates to a total score of 3 + 4 = 7. Local guidelines categorise PCa risk using the GS as follows; (i) GS ≤ 6: low-risk, (ii) GS = 7: intermediate-risk and (iii) GS ≥ 8: high-risk [4, 5]. Patients with a high-risk GS have a poorer prognosis with an increased risk of metastatic progression and death [6]. For these patients, the PCa mortality risk is 60 to 87% compared to between 42 and 70% for an intermediate-risk GS [6].

Across the National Health Laboratory Service (NHLS), a laboratory information system (LIS) is used to record, manage, and store patient laboratory reports and related demographic health data [7, 8]. This LIS documents all processes within the laboratory workflow including sample registration, test order generation, tracking orders and reporting results [7, 8]. For AP reporting, the assigned pathologist voice-records the biopsy narrative report for electronic capture by data typists. These narrative AP reports are not standardised and are pathologist dependent in terms of patient history, pathological tumour/biopsy description and language used. As a result, these are irregularly reported text-intensive patient reports. Table 1 provides an example of a semi-structured narrative biopsy report that includes the headings clinical history, macroscopy and pathological diagnosis (highlighted in bold).

**Table 1:** Example of the semi-structured narrative prostate biopsy report

Category	Biopsy report
Biopsy report	EPISODE NUMBER: ABC1234 **CLINICAL HISTORY**: A 67 YEAR OLD MALE PATIENT WITH A PSA OF 7.9 UG/L. PROSTATE BIOPSIES HAVE BEEN DONE. **MACROSCOPY**: SIXTEEN CORES OF TISSUE, THE LONGEST MEASURING 15MM AND THE SHORTEST MEASURING 7MM. **PATHOLOGICAL DIAGNOSIS**: PROSTATE CORE BIOPSIES SHOWING THE FOLLOWING FEATURES: AN INVASIVE PROSTATIC ADENOCARCINOMA. TWO CORES ARE INVOLVED AND < 5% OF THE TISSUE. GLEASON 4, 3. PERINEURAL AND LYMPHOVACULAR INVASION ARE NOT IDENTIFIED. IMMUNOHISTOCHEMISTRY: IN THE PRESENCE OF ADEQUATE POSITIVE CONTROLS, IMMUNOHISTOCHEMICAL STAINS HAVE BEEN DONE AND THE FOLLOWING RESULTS OBTAINED: P63 AND CK5/6: BASAL CELLS ARE NOT DEMONSTRATED IN THE ATYPICAL GLANDS

The narrative biopsy report included the headings clinical history, macroscopy and pathological diagnosis

PSA: prostate specific antigen MM: millimetre P63: Protein 63 CK5/6: Cytokeratin 5/6

The GS is reported as embedded text within semi-structured narrative biopsy reports in alpha, numeric as well as alphanumeric formats. As a result, the GS score could be captured in a variety of patterns based on the local AP practices. For example, a GS of 4 + 4 = 8 may be captured as: (i) 4 + 4 = 8, (ii) 8 (4 + 4), (iii) 4;4 and (iv) major 4, minor 4.

Spacic et al. have reported that the linear structure of the GS makes it amenable to modelling using regular expressions [9]. In contrast, various cancer specific vocabularies and classification systems as well as ontologies have been used with text mining to extract structured information from narrative biopsy reports [9]. These vocabularies and ontologies work well with coding systems such as International Classification of Diseases for Oncology (ICD-O-3), Systemized Nomenclature of Medicine (SNOMED) Clinical Terms (CT) and International Classification of Diseases Tenth Revision (ICD-10) for example [9]. Such vocabularies and ontologies do not exist for the GS. As a result, the manual coding of the GS is time-consuming resulting in a paucity of local data describing late presentation in South Africa.

Globally, artificial intelligence (AI) has been used to automate decision making through mimicking human cognitive function by using mathematical, statistical, logical, and computer programming approaches [10, 11]. The AI model can be trained using existing data and applied to new data to automate decisions [11]. AI can also be applied to semi-structured healthcare data using techniques such as natural language processing (NLP) [10]. This is achieved by employing computational techniques to extract semantic meaning from text [12, 13]. In essence, these NLP procedures convert text to machine-readable structured data [10]. This includes computational approaches such as tokenisation that help to identify words and punctuations within a sentence [13]. In summary, NLP can be used to extract clinical information from unstructured data to supplement and enrich structured medical data [10].

There is a need to develop automated algorithms that can extract the GS from narrative prostate biopsy reports. The objective of our study was to explore the use of text mining techniques to extract the predictive GS from narrative prostate biopsy reports.

## Methods

All methods were carried out in accordance with relevant guidelines and regulations of the Human Research Ethics Committee (Medical) at the University of the Witwatersrand (Faculty of Health Sciences).

### Experimental design

We did not perform any experiments since the algorithm was not trained or optimised through various iterations. The only experiment that was conducted is described in the methods sections below.

### Text mining algorithm development

**Fig. 1:**
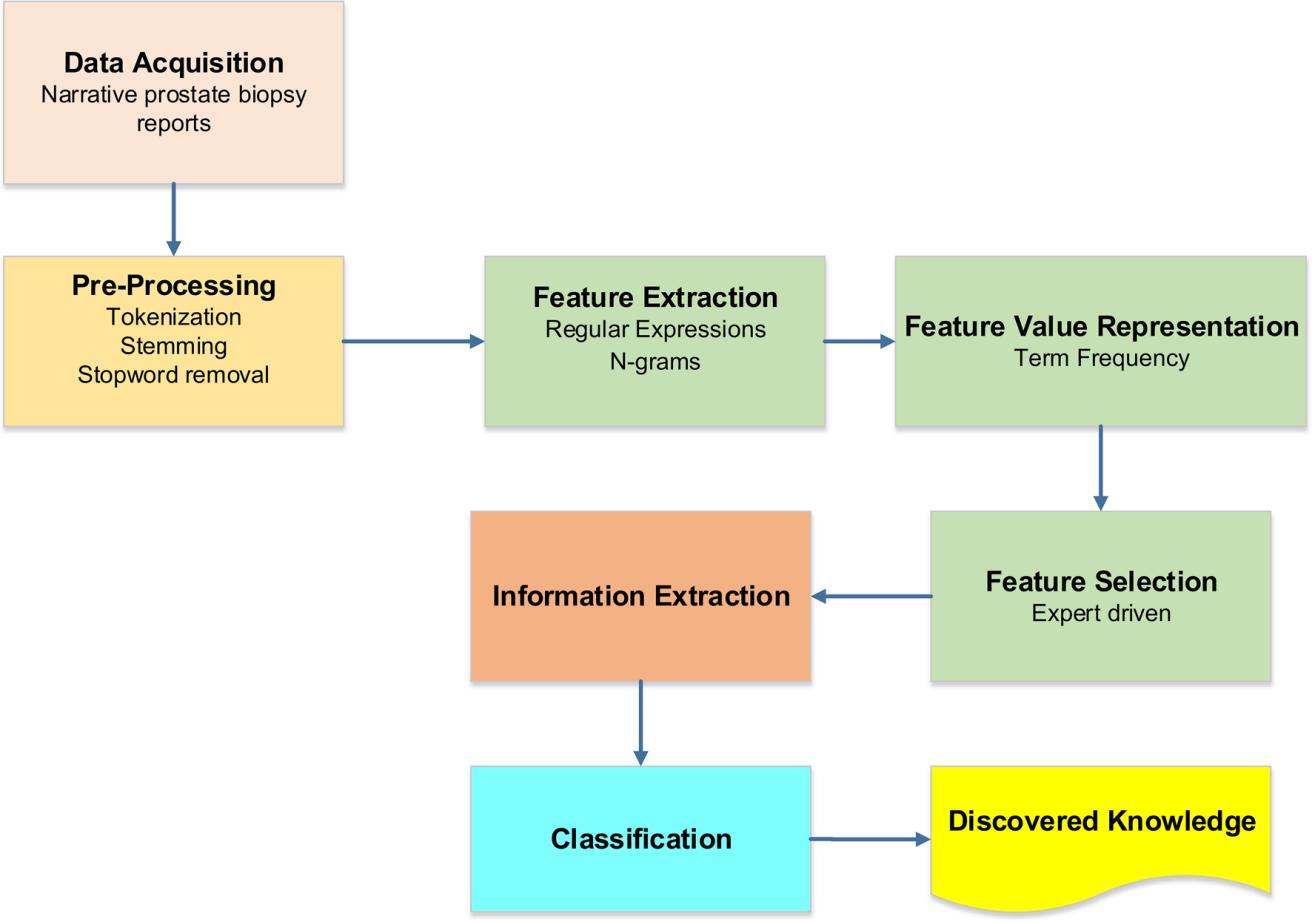
Diagram describing the logical processes used to analyse the raw narrative prostate biopsy report to generate the discovered knowledge. The steps were as follows: (i) data acquisition (ii) pre-processing and (iii) feature extraction, (iv) feature value representation, (v) feature selection, (vi) information extraction (vii) classification and (viii) discovered knowledge

We used the Python Spyder integrated development environment (IDE) for the development of the text mining algorithm because of its robustness in advanced editing, debugging, profiling, data exploration and interactive execution [14, 15]. An IDE is software that is used to build and develop applications. The Python code for this algorithm has been uploaded on GitHub (https://github.com/VictorO2/text-mining-gleason-score). The following Python modules were imported: (i) os, (ii) pandas, (iii) time, (iv) matplotlib, (v) seaborn, (vi) WordCloud and, (vii) Natural Language Toolkit (NLTK). We followed the text mining pipeline as depicted in the flowchart below (Fig. 1). The logical steps for the text mining algorithm were as follows: (i) data acquisition (ii) pre-processing, (iii) feature extraction, (iv) feature value representation, (v) feature selection, (vi) information extraction, (vii) classification, and (viii) discovered knowledge.

### Data acquisition

We extracted all prostate biopsies performed for men aged ≥ 30 years between 1 January 2006 and 31 December 2016 that were referred to the NHLS for pathology evaluation in the Gauteng province, South Africa. Two data sets were extracted from the national laboratory data repository that houses LIS collated patient laboratory reports. The narrative prostate biopsy reports are captured as free-text in the LIS and stored in the national laboratory data repository. The Systematised Nomenclature of Medicine (SNOMED) clinical terms (CT) dataset was used to develop lookup tables to identify biopsies with an adenocarcinoma histological finding (n = 8201) [16]. Once the biopsies with PCa were identified (adenocarcinoma histological findings with a reported GS), we extracted a random sample of 1000 biopsies using Microsoft Excel (Redmond, Washington, USA) [17]. We chose a random sample as we did not want to select biopsies that were reported in a similar fashion from one laboratory.

To evaluate the text mining algorithm, we also randomly extracted 1000 prostate biopsy narrative reports with a PCa diagnosis that were submitted from private sector laboratories to the National Cancer Registry (NCR) (referred to as the validation dataset). These narrative reports are generated by various private sector pathology practices and could be used to validate the algorithm. We received only the narrative biopsy reports.

For both datasets, the GS were manually coded by two experts. Manual coding was required as the GS is not extracted by the NCR and is embedded within the narrative report. Following this, a random sample of 369 biopsies were independently verified to validate the manual coding.

### Pre-processing

We used pre-processing to ensure that the narrative biopsy reports were in a machine-readable format. The first step was to convert the narrative reports to a document format (also referred to as a corpus). A corpus is defined as large and unstructured text. This is required to convert the narrative reports into a structured format that is required for text mining [14, 18, 19]. Next, the data cleaning process involved using the NLP tokenization, stopwords removal and stemming techniques [15, 19]. We used tokenization to condense the streams of text into smaller meaningful elements (called tokens) that comprised of words, phrases and symbols. For example, the words ‘do not stop’ would result in 3 tokens (do-not-stop). We employed stemming to create various variants of words into a common representation known as the stem. Stemming takes words or a set of words to their root form, e.g., root of “gleasen” is “gleason”. We also standardised the word Gleason, major, minor, score, etc. Finally, we used the NLTK toolkit stop words to filter and remove irrelevant words before text processing, e.g. the, is, at, etc. This removed all possible English stopwords. We also converted text to lowercase for standardisation.

### Feature extraction

As part of feature extraction, we used an expert rule-based approach. The experts manually crafted the regular expressions. We extracted features of interest from narrative prostate biopsy reports. We used regular expressions representative of the GS target feature such as “gleason”, “Gleason”, “GLEASON”, “Gleeson”, etc. for feature extraction. Regular expressions can be used to define a sequence of characters that are associated with a feature. Each of these text patterns can be used as a rule-based approach to extract a feature. Similar approaches have been described by Napolitano and Spacic et al.[9, 20]. Next, we used N-grams as our feature extraction strategy to extract the major and minor Gleason scores. We created unigrams, bigrams, trigrams and quadgrams which generated these scores. N-grams is a methodology that looks at sequences of words which are most occurring depending on the size of n, i.e. sequence of n words. N-grams are a set of co-occurring terms that were reported in a sentence or paragraph in the corpus [21, 22]. For example, when n = 1 (unigram) this represents single words in a sentence [22]. Similarly, when n is equal to 2 (bigram), 3 (trigram) or 4 (quadgram) this is represented as two, three and four words in a sentence respectively [22]. From the N-grams generated, we extracted the GS feature for each biopsy. The N-gram feature extraction output is provided for a sample of biopsies (Table 2).

**Table 2:** N-grams feature extraction output for a sample of biopsies

[‘major 4 minor 5’, ‘4 + 5’]	[‘4 + 4’, ‘4 + 4’]	[‘3 + 3’, ‘3 + 3’]	[‘4 + 4’, ‘4 + 4’]	[‘major 4 + minor 3’]	[‘4 + 5’]
[‘major 4 minor 5’, ‘4 + 5’]	[‘major 4 minor 4’]	[‘4 + 4’, ‘4 + 4’]	[‘4 + 4’, ‘4 + 4’]	[‘major 5 minor 4’]	[‘major 3 minor 5’]
[‘3 + 3’, ‘3 + 3’]	[‘2 + 2’]	[‘3 + 4’, ‘3 + 4’]	[‘3 + 2’, ‘3 + 3’, ‘3 + 3’]	[‘major 4 + minor 5’]	[‘major 3 minor 4’]
[‘3 + 5’]	[‘3 + 3’, ‘3 + 3’]	[‘2 + 2’, ‘2 + 2’, ‘2 + 2’]	[‘3 + 5’, ‘3 + 5’]	[‘4 + 3’]	[‘3 + 5’, ‘major 4 + minor 5’]
[‘major 4 minor 5’]	[‘4 + 3’, ‘4 + 3’]	[‘3 + 2’, ‘3 + 5’, ‘3 + 5’]	[‘3 + 3’]	[‘major 5 minor 4’]	[‘major 3 minor 5’]
[‘4 + 3’]	[‘2 + 3’, ‘2 + 3’]	[‘2 + 2’]	[‘3 + 2’, ‘3 + 2’]	[‘major 5 + minor 4’]	[‘major 5 + minor 4’]
[‘major 4 minor 3’]	[‘3 + 3’, ‘3 + 3’]	[‘4 + 4’, ‘4 + 4’]	[‘3 + 4’, ‘3 + 4’]	[‘major 3 minor 4’]	[‘major 4 minor 5’]
[‘3 + 2’, ‘3 + 2’]	[‘4 + 3’, ‘4 + 3’]	[‘2 + 3’, ‘3 + 4’, ‘3 + 4’]	[‘4 + 3’, ‘4 + 3’]	[‘major 5 minor 5’]	[‘major 3 minor 4’]
[‘major 4 + minor 3’]	[‘3 + 3’, ‘3 + 3’]	[‘4 + 4’, ‘4 + 4’]	[‘3 + 3’, ‘3 + 3’]	[‘major 5 minor 4’]	[‘major 4 minor 5’]
[‘5 + 5’, ‘5 + 5’]	[‘3 + 3’, ‘3 + 3’]	[‘5 + 4’, ‘5 + 4’]	[‘4 + 5’]	[‘major 3 minor 3’]	[‘major 5 minor 3’]
[‘major 3 minor 4’]	[‘major 5 + minor 5’]	[‘major 5 minor 4’]	[‘major 3 minor 5’]	[‘major 4 minor 5’]	

### Feature value representation

**Fig. 2:**
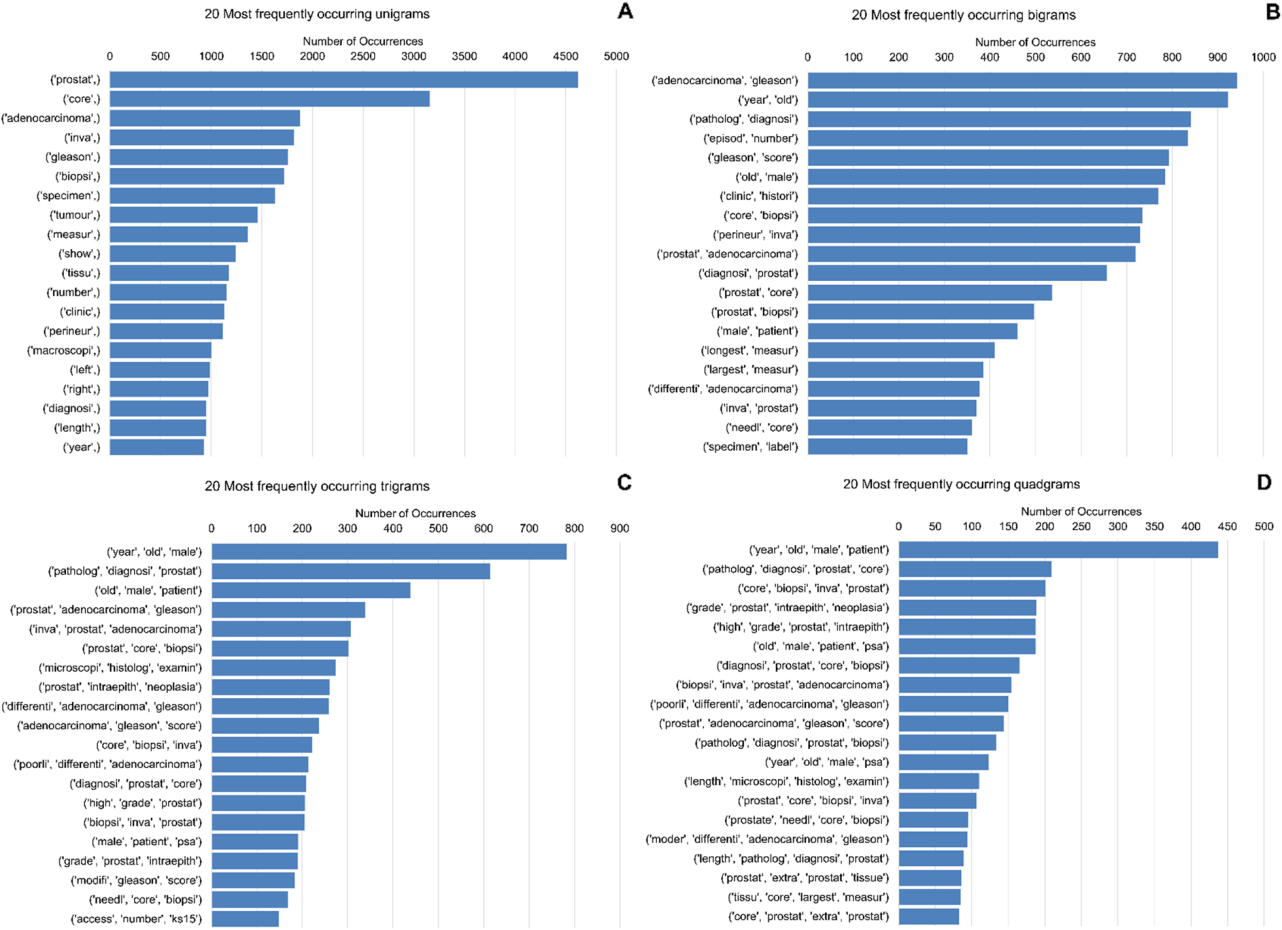
Horizontal bar graph depicting the top twenty occurring unigrams (**A**), bigrams (**B**), trigrams (**C**) and quadgrams (**D**). The number of occurrences is displayed on the x-axis

For feature representation, we created a document term matrix using term frequency. This was used to transform the document into a numeric feature vector space. We reported the twenty most frequently occurring unigrams, bigrams, trigrams and quadgrams as horizontal bar graphs (Fig. 2).

### Feature selection

For feature selection, we used pathologists (experts) who identified key words that could be used to identify the features of interest in the narrative prostate biopsy reports. As part of expert driven feature selection, we used these key words in the algorithm to select the following features: (i) episode number, (ii) major score, (iii) minor score, (iv) total score and (v) combined score. Because we used expert driven feature selection, we only chose relevant features and reduced the feature space (without using dimensionality reduction). Reducing the number of features selected would improve the model performance. Even though the feature space was reduced, there was no loss of information [23].

### Information extraction

Information extraction is used to select specific entities and relationships of interest [9]. For information extraction, we manipulated the N-grams output to extract the numerical value of the major and minor scores. This was an automated process where the Gleason score was identified from N-grams by the algorithm. This was achieved by splitting the major and minor scores from the N-grams. Next, we removed all non-numerical characters to remain with only the scores. The scores were then converted to numbers. Next, we calculated the total score and reported the GS in a standardised format, e.g., 4 + 4 = 8.

### Classification

We classified biopsies into the three risk categories: (i) low (≤ 6), (ii) intermediate (7) and (iii) high-risk (≥ 8) based on local guidelines [5]. The classification process was automated using a rule-based approach and implemented within the algorithm.

### Discovered knowledge

The discovered knowledge included the episode number, major score, minor score, total score, standardised GS and risk category. For each biopsy, the algorithm extracted a single row of structured data. From the narrative biopsy report depicted in Table 1, the following discovered knowledge was reported: (i) ABC1234, (ii) 4, (iii) 3, (iv) 7, (v) 4 + 3 = 7 and (vi) intermediate.

### Text mining algorithm evaluation

A confusion matrix (also known as a sensitivity/specificity analysis) was used to compare the text mining algorithm extracted against the manually coded values [24]. The confusion matrix consists of four values: (i) True Positives (TP): correctly extracting the GS, (ii) True Negatives (TN): correctly extracting a biopsy without a GS, (iii) False Positive (FP): falsely extracting a GS and (iv) False Negative (FN): falsely extracting the manually coded GS [24]. The precision and recall are calculated using these four values as follows: (i) $\frac{TP}{TP+FP}$ and (ii) $\frac{TP}{TP+FN}$ respectively. Precision and recall are similar to positive predictive value (PPV) and sensitivity respectively. The F-score is the harmonic mean of precision and recall and is calculated using the formula $\frac{2*(Recall *Precision)}{(Recall+Precision)}$. The manually coded values were assumed to be the gold standard, i.e. exact match. Therefore, we reported the data as ‘Exact Match: Yes’ and ‘Exact Match: No’ for both the predicted and manually coded values.

### Statistical analysis

We reported the top ten GS alpha, numeric and alphanumeric reporting formats as a table, i.e., how they were captured in the narrative prostate biopsy report. We also reported the top five GS reported, with the remaining scores categorised as ‘Others’. The percentage of a top five GS categorised as high-risk (≥ 8) is also indicated. As we reported data for a multi-class problem, we reported the frequencies for the predicted and manually coded values for a low, intermediate and high-risk GS as a table. Next, we calculated the macro averaged F-score (F-score for each GS risk category added up and then divided by the number of measurements) [25].

## Results

The random sample taken from 1000 prostate biopsies showed no manually coded GS misclassification errors for both datasets.

### Text mining algorithm performance

For 1000 narrative biopsies, the text mining algorithm extracted the GS in a time of under 10 min for both the study and validation datasets. The word cloud before and after cleaning revealed which text was more important. After using trigrams and quadgrams, the algorithm had both extracted all the GS and exhausted the sequence of words. Therefore, there was no need to use more than four grams, i.e., we had exhausted all word combinations. Our dataset was also small and logical extraction of n-Grams could only go up to four. With a larger corpus, we would have to explore using more n-Grams, e.g., 10. The term frequency analysis revealed that the Gleason score appeared as the fourth most common term for unigrams (n = 1754). For the bigrams, the term Gleason score appeared in position one (n = 942) and four (n = 793). Similarly, the Gleason score appeared four times in trigrams compared to thrice for quadgrams.

### Text Mining precision and recall

The first text mining algorithm output reported an F-score of 0.99 (recall: 0.98 precision: 1.00) (Table 3). On manual inspection of the N-grams (Table 2), we identified that two different GS were reported in both the clinical history and pathological diagnosis for 16 biopsies (example ‘3 + 2’, ‘3 + 3’, ‘3 + 3’ in Table 2). The algorithm was updated to report the latter GS resulting in an F-score of 1.00 (recall: 1.00 and precision: 1.00). The text mining algorithm was tested on the validation dataset and reported an F-score of 0.99.

**Table 3:** Performance of the text mining algorithm to automate the extraction of the Gleason score from narrative prostate biopsy narrative reports

	Manual coding	
	Exact Match: Yes	Exact Match: No
*First algorithm output*		
Predicted		
Exact Match: Yes	984	0
Exact Match: No	16	0
Precision = 1.00		
Recall = 0.98		
F-score = 0.99		

	Manual coding	
	Exact Match: Yes	Exact Match: No
*Updated algorithm output*		
Predicted		
Exact Match: Yes	1000	0
Exact Match: No	0	0
Precision = 1.00		
Recall = 1.00		
F-score = 1.00		

	Manual coding	
	Exact Match: Yes	Exact Match: No
*Validation dataset output*		
Predicted		
Exact Match: Yes	988	0
Exact Match: No	12	0
Precision = 1.00		
Recall = 0.988		
F-score = 0.99		

A contingency table was used to compare the manually coded and algorithm predicted values. We reported the precision, recall and F-score reported for the first and updated text mining algorithm output as well as for the validation dataset.

### Gleason score formats reported

We identified ten different GS reporting formats (Table 4). The variations in reporting included: (i) use of both the equal sign as well as the word equals, (ii) use of brackets, (iii) spelling of major and minor (for example using the word major and pattern), (iv) use of both the words and symbols (plus versus +) and (v) use of colons and commas to separate major and minor scores.

**Table 4:** Different Gleason score formats reported for the study

#	Extracted score	As reported in the biopsy report
1	5 + 4 = 9	5, 4
2	5 + 4 = 9	5 PLUS 4 EQUALS 9
3	3 + 3 = 6	3 + 3 = 6 OR 3 + 3
4	3 + 5 = 8	MAJOR PATTERN 3, MINOR PATTERN 5
5	4 + 3 = 7	MAJOR PATTERN: 4/5 MINOR PATTERN: 3/5
6	4 + 3 = 7	MAJOR 4 PLUS MINOR 3 EQUALS 7
7	5 + 3 = 8	SCORE 8 (MAJOR 5; MINOR 3)
8	3 + 4 = 7	7 (3 + 4)
9	4 + 3 = 7	(4 + 3) = 7
10	3 + 4 = 7	3 (MAJOR) + 4 (MINOR) = 7/10

The clean extracted score reported, and the original value reported in the prostate biopsy report is indicated for the study dataset

### Gleason score frequency analysis

The most commonly reported GS were 5 + 4 = 9, 3 + 3 = 6 for 17.6% (n = 176) and 17.5% (n = 175) of biopsies respectively (Table 5). There were 164 biopsies with a 4 + 3 = 7 score (16.4%). A 3 + 4 = 7 and 4 + 4 = 8 GS was reported for 14.7% (n = 147) and 14.2% (n = 142) biopsies respectively. The remaining GS comprised 19.4% (n = 196) of biopsies. A high-risk GS was reported for 31.8% of biopsies. For the validation dataset, the most commonly reported GS were: (i) 3 + 3 = 6 (37.7%), (ii) 3 + 4 = 7 (19.4%), (iii) 4 + 3 = 7 (14.9%) and (iv) 4 + 4 = 8 (10.0%) and (v) 4 + 5 = 9 (7.4%). A high-risk GS was reported for 17.4% of biopsies.

**Table 5:** The table reported the frequency for the top five reported Gleason scores with the remaining values grouped and reported as “Others”

No	Study dataset			Validation dataset		
	Gleason score	n =	%	Gleason score	n =	%
1	5 + 4 = 9	176	17.6	3 + 3 = 6	377	37.7
2	3 + 3 = 6	175	17.5	3 + 4 = 7	194	19.4
3	4 + 3 = 7	164	16.4	4 + 3 = 7	149	14.9
4	3 + 4 = 7	147	14.7	4 + 4 = 8	100	10.0
5	4 + 4 = 8	142	14.2	4 + 5 = 9	74	7.4
6	Others	196	19.6	Others	106	10.6
Total		1000	100	Total	1000	100
High-Risk GS ≥ 8		318	31.8	High-Risk GS ≥ 8	174	17.4

Data is reported for this study as well as for the separate dataset

GS: Gleason score

### Gleason risk category analysis

For a low-risk GS, there were 199 predicted and 193 manually coded values (difference of 6), with an F-score of 0.98 (Table 6). Similarly, for an intermediate and high-risk GS a difference of 3 was reported for both groups with an F-score of 1.00 and 1.00 respectively. The macro-average F-score was 0.99 and macro recall and precision were 1.00 and 0.99 respectively.

**Table 6:** Comparison of low, intermediate and high-risk Gleason scores for the predicted and manually coded values

GS risk category	Predicted	Manually coded	F-score
Low-risk GS (≤ 6)	199	193	0.98
Intermediate-risk GS (7)	311	314	1.00
High-risk GS (≥ 8)	490	493	1.00
*p*-value^&^			0.9439
Macro-average F-score			0.99
Macro recall			1.00
Macro precision			0.98

The macro-average F-score is reported

GS: Gleason score

^&^Alpha value of 0.05 used to assess significance

## Discussion

The objective of our study was to explore the use of text mining techniques to extract the GS from irregularly reported text-intensive narrative prostate biopsy reports. The first text mining algorithm output reported that 16/1000 biopsies GS (1.6%) was inaccurately predicted. On inspection of the N-grams, we identified that these biopsies had two reported GS, once in clinical history and again in the biopsy report. We amended the text mining algorithm, resulting in all 1000 GS accurately extracted with an F-score of 1.0. The attained F-score suggests that our feature engineering process was effective as we managed to pull out discriminative features that were most representative of our dataset. The text mining algorithm was further evaluated against a validation dataset, with good overall accuracy and precision (F-score of 0.99). The F-score reported for both datasets is similar to a Perl routine that also used regular expressions to extract the GS [20]. Similar approaches using regular expressions have been reported by two other studies [9, 20].

Our findings reveal that despite the variability in the GS reporting, the text mining algorithm was able to extract the GS. This indicates that in settings with different AP reporting styles, the text mining algorithm would still be able to extract the required features. This is a promising finding that indicates that the text mining algorithm can handle varying reporting formats.

We noted a difference in the top five reported GS for our study and the validation dataset. We reported a high-risk GS for 31.8% of biopsies compared to 17.4% for the validation dataset. This indicates that late presentation differed between the public and private sector. This could be explained by the racial variation in medical aid coverage [26]. A limitation of this study is the small sample sizes.

As we reported data for a multi-class problem and compared the predicted and manually coded values categorised as low, intermediate and high-risk [5]. The analysis revealed an acceptable macro-averaged F-score indicating that the text mining algorithm was able to accurately classify the GS risk category.

Our findings indicate that the text mining algorithm could be used to reliably extract the GS from laboratory data in similar settings. Given the paucity of local PCa data, this algorithm would make it easier to conduct studies for larger sample sizes. This would be achieved by implementing the text mining algorithm as an API [27]. The text mining algorithm code can be packaged as an executable application that can be applied to routinely extract data from narrative laboratory reports. Such an approach could be used to facilitate the generation of important predictive clinical information for PCa using any LIS based data to derive both retrospective and prospective health information. This would dramatically improve the availability of the GS data for local studies and routine surveillance.

While our study was conducted for only English narrative reports, this approach could be extended to non-English data. In an African context, it would be important to extend these approaches specifically to Arabic, French, Portuguese, Spanish and Kiswahili [28]. Natural language processing could be used to convert these narrative reports into a common English language as demonstrated by Delegér et al.for French [29]. As such, once this is done the processing using the existing text mining algorithm would be possible. A limitation of our study is that the text mining algorithm was not applied to all 8201 narrative reports with PCa. The objective this study was to pilot the use of a text mining algorithm for a sample of biopsies. We are looking at applying the text mining algorithm to national prostate biopsy narrative reports as the next step.

The text mining tools employed in our study could be used to extract clinical information for other cancers of public health interest. For example, breast cancer biopsies are graded using the modified Bloom and Richardson system [30]. This grading system is similar to the GS as it reports the cytology, tubule formation, nuclear pleomorphism, and the mitotic count to determine the grade (I, II or III) [30]. Therefore, the techniques employed in our study could be applied to other narrative laboratory data such as immunophenotyping, fluorescence in situ hybridization and leukaemia reports, to extract important clinical, diagnostic and predictive information.

Furthermore, to address the remaining 12% of biopsies without a SNOMED CT code, our text mining algorithm could be supplemented by machine learning (ML) to extract an adenocarcinoma histological finding in an automated fashion [9]. This has the potential to offer near real-time cancer registry type information removing the need for manual coding [31]. This would also dramatically reduce the time from reporting to generating surveillance data. In addition, the extraction of the GS would make it possible to better assess trends in late presentation.

In addition to ML approaches, we would also recommend using deep learning approaches. Deep learning is composed of multiple processing layers that learn representations of data with multiple levels of abstraction [32]. This approach has dramatically improved AI approaches for visual object recognition and object detection [32]. Deep learning models are able to extract information from large datasets and will continue to improve the knowledge discovery as more data is generated [33]. This enables deep learning to outperform classical ML approaches [33]. One of the benefits is that deep learning can extract the feature without the need for supervision required by ML. A good example is representation learning, a deep learning approach that automatically discovers the representations needed for detection or classification from raw data [32].

Once these ML and deep learning algorithms have been developed, it would be possible to move the extraction of an adenocarcinoma histological finding with the GS to a cloud service. This would make it possible for narrative prostate datasets to be uploaded using an internet connection and the extracted knowledge delivered as a data extract. Similar approaches have been demonstrated for breast cancer [34]. This has the potential for cancer registries across Africa to load their narrative data and obtain coded data for incidence and late presentation surveillance activities.

## Conclusion

Our study has shown that a text mining algorithm can be used to extract the predictive GS from narrative biopsy reports. This could also be used to better assess late presentation by extracting the GS in an automated fashion. These tools have the potential to describe PCa in an African context with a paucity of data. This approach is applicable to other cancers of public health interest. Furthermore, ML and deep learning approaches should be investigated to replicate results shown for the SNOMED CT lookup tables to address data gaps. These could be used to reduce the delays in the publication of cancer registry data. These algorithms could be moved to a cloud service to extend automated PCa surveillance data generation across Africa.

## Data Availability

The datasets generated and/or analysed during the current study are not publicly available as the authors do not have permission to share them. Consent to access the datasets should be directed to naseem.cassim@wits.ac.za.

## Abbreviations

AI: Artificial Intelligence
AP: Anatomical Pathology
ASIR: Age-standardised incidence rate
CSV: Comma separated value
CT: Clinical terms
FN: False negative
FP: False positive
GS: Gleason score
ICD: International Classification of Diseases
ICD-0-3: International Classification of Diseases for Oncology–3rd Edition
ICD-10: International Classification of Disease–Tenth revision
IDE: Integrated development environment
LIS: Laboratory Information System
ML: Machine Learning
NCD: Non-Communicable Diseases
NCR: National Cancer Registry
NHLS: National Health Laboratory Service
NLP: Natural Language Processing
NLTK: Natural Language Toolkit
PCa: Prostate Cancer
PPV: Positive predictive value
PSA: Prostate specific antigen
SNOMED: Systemized Nomenclature of Medicine
TN: True negative
TP: True positive
